# Identifying bottlenecks in the iron and folic acid supply chain in Bihar, India: a mixed-methods study

**DOI:** 10.1186/s12913-018-3017-x

**Published:** 2018-04-12

**Authors:** Amanda S. Wendt, Rob Stephenson, Melissa F. Young, Pankaj Verma, Sridhar Srikantiah, Amy Webb-Girard, Carol J. Hogue, Usha Ramakrishnan, Reynaldo Martorell

**Affiliations:** 1 UniversitätsKlinikum Heidelberg, Institut für Public Health, Im Neuenheimer Feld 324, 69120 Heidelberg, Germany; 2 Nutrition and Health Sciences, Division of Biological and Biomedical Sciences, Emory University, 1462 Clifton Rd. Suite 314, Atlanta, GA 30322 USA; 3 Department of Health Behavior and Biological Sciences, University of Michigan School of Nursing, University of Michigan, 400 North Ingalls Building, Ann Arbor, MI 48109 USA; 4 Hubert Department of Global Health, Rollins School of Public Health, Emory University, 1518 Clifton Rd. NE, Atlanta, GA 30322 USA; 5 Integrated Family Health Initiative, CARE Bihar, 2nd Floor, 10, IAS Colony, Kidwaipuri, Patna, Bihar 800 001 India; 6 Department of Epidemiology, Rollins School of Public Health, Emory University, 1518 Clifton Rd. NE, Atlanta, GA 30322 USA

**Keywords:** Maternal health, Evaluation, Antenatal care, Iron and folic acid, Qualitative, Supply chain

## Abstract

**Background:**

Maternal anaemia prevalence in Bihar, India remains high despite government mandated iron supplementation targeting pregnant women. Inadequate supply has been identified as a potential barrier to iron and folic acid (IFA) receipt. Our study objective was to examine the government health system’s IFA supply and distribution system and identify bottlenecks contributing to insufficient IFA supply.

**Methods:**

Primary data collection was conducted in November 2011 and July 2012 across 8 districts in Bihar, India. A cross-sectional, observational, mixed methods approach was utilized. Auxiliary Nurse Midwives were surveyed on current IFA supply and practices. In-depth interviews (*n* = 59) were conducted with health workers at state, district, block, health sub-centre, and village levels.

**Results:**

Overall, 44% of Auxiliary Nurse Midwives were out of IFA stock. Stock levels and supply chain practices varied greatly across districts. Qualitative data revealed specific bottlenecks impacting IFA forecasting, procurement, storage, disposal, lack of personnel, and few training opportunities for key players in the supply chain.

**Conclusions:**

Inadequate IFA supply is a major constraint to the IFA supplementation program, the extent of which varies widely across districts. Improvements at all levels of infrastructure, practices, and effective monitoring will be critical to strengthen the IFA supply chain in Bihar.

**Electronic supplementary material:**

The online version of this article (10.1186/s12913-018-3017-x) contains supplementary material, which is available to authorized users.

## Background

Worldwide 1.62 billion people are estimated to have anaemia [1]. A recent analysis estimates that anaemia affects 38% of pregnant women globally [2]. In Bihar, India, even greater proportions of ever-married (60%) and pregnant women (58%) are anaemic, higher than national prevalence estimates (53% and 50%, respectively) [3, 4]. Women with maternal anaemia are more likely to deliver preterm and have low birth weight offspring [5] with low foetal iron stores leading to impaired cognitive development [6]. Severe anaemia can lead to complications during delivery including increased blood loss, cardiac failure, and maternal mortality [5]. Fortunately, oral iron supplementation has been recognized as an effective prevention and treatment of iron deficiency anaemia [7, 8].

In 1970, the Government of India established the National Nutritional Anaemia Prophylaxis Programme to address widespread anaemia, targeting high risk groups for iron supplementation. The programme was expanded in 1991 to universal supplementation of pregnant and lactating women. Current recommendations include a daily dose of 100 mg elemental iron for 100 or more days from 14 to 16 weeks of pregnancy until the 3rd month post-partum [9]. Unfortunately, despite these guidelines, high anaemia prevalence persists [5]. In Bihar, only 10% of women reported consuming IFA for at least 100 days during their last pregnancy, despite 80% reporting registered pregnancies, implying at least one at least one antenatal care visit [4]. Barriers to IFA intake by pregnant women in India and other low and middle income countries have included gastrointestinal side effects [7, 10–13], lack of comprehensive counselling by healthcare providers [14–17], IFA negatively seen as medicine [14], and distrust of government IFA or freely available IFA [14, 17]. Supply issues have also been recognized as a barrier to IFA adherence [14, 16–18]. In Bihar, this seems likely as infrastructure, personnel, and supply chain challenges have been highlighted as major constraints of the health system. A 2009 report found Bihar’s health sub-centres to be lacking in all existing amenities and functionality indicators measured [19]. In 2007–08, a state-wide survey found only 56% of primary health centres and 6% of sub-centres had at least 60% of required drugs present. In particular, IFA had been out of stock 10 days of the previous month in 78% of surveyed health sub-centres [20]. More recent reports from 2014 to 2016 continue to highlight drug shortages and procurement challenges as key issues to address in the Bihar health system [21–23]. To our knowledge, no studies to date have set out to comprehensively describe the IFA supply chain in India, characterize potential bottlenecks to access and availability, and identify feasible and contextually relevant solutions.

Our objective was to explore the current status of the Government of Bihar’s IFA supply and distribution system to identify bottlenecks that may contribute to insufficient or inconsistent supply. Based on knowledge gained from participant responses, we offer practical recommendations that can be implemented by the government and collaborating organizations.

## Methods

In November 2011 and July 2012 we conducted a cross-sectional, mixed methods study to characterize IFA supply chain protocols and procedures across 8 districts. These were the initial focus districts of a CARE Bihar (a non-governmental humanitarian organization) project titled the Integrated Family Health Initiative conducted with support from the Bill and Melinda Gates Foundation. This programme supports the Bihar government to improve maternal and child health outcomes by increasing delivery, uptake, and utilization of family health services [24]. Our study included qualitative in-depth interviews with key players in the IFA supply chain and surveys distributed to Auxiliary Nurse Midwives (ANMs).

From both qualitative and quantitative data collection, we developed a description of the IFA supply chain and ANM receipt and distribution. From qualitative data, we present supply chain bottlenecks identified by interview participants.

### Qualitative in-depth interviews

#### Participants

In total, 59 in-depth interviews were conducted with health workers at state, district, block, health sub-centre, and village levels. Officials in the Health Department, National Rural Health Mission (NRHM), and Integrated Child Development Scheme (ICDS), were included (Table 1). ANMs, Accredited Social Health Activists (ASHAs), and Anganwadi Workers (AWWs) are classified as frontline workers (FLWs) as they are responsible for IFA distribution to beneficiaries in the field (see Additional file 1, Additional file 2, Additional file 3, Additional file 4, Additional file 5, Additional file 6, Additional file 7 and Additional file 8 for interview guides).

**Table 1:** Qualitative In-Depth Interviews Conducted

Supply Chain Level	Participants Interviewed	Total Interviews (n)
*State*	State Health Society Official	1
*District*	Civil Surgeon	20
	District Programme Manager	
	District Storekeeper	
	District Hospital Manager	
*Block*	Medical Officier in Charge	14
	Block Health Manager	
	Block Community Mobilizer	
	Pharmacist	
	Clerk	
*Health Sub-Centre*	Auxiliary Nurse Midwife	13
*Village*	Accredited Social Health Activist	11
	Anganwadi Worker	

Interviews were requested by CARE staff. Frontline workers were recruited based on a priori selection of coverage areas. Initially, blocks near to and far from the district capital, as defined by CARE staff, were chosen to add variation. Later, blocks were selected randomly with modifications due to time constraints.

#### Data collection

In-depth interview guides, drafted in English, focused on respondents’ perceived roles in IFA receipt and distribution, IFA need estimation, and trainings. Guides were constructed taking into account relevant literature [16] and iterative discussions with co-authors and CARE personnel. One bilingual research assistant used English guides to conduct interviews. Others required interview guides translated into Hindi. Quality and accuracy were verified through review of guides and recordings by external bilingual researchers. All interviews were conducted in Hindi and lasted 30–60 min. Research assistants were trained in qualitative research techniques and debriefed daily with the primary author. Interviews were recorded along with written notes and observations. The majority of recordings were transcribed directly into English by a bilingual researcher. Ten additional interviews, collected in July 2012, were transcribed into Hindi and translated into English in a two-step process. On a subset of transcripts, quality checks were completed by comparison of recordings to transcripts by an external bilingual researcher.

#### Data management and analysis

All transcripts were de-identified for analysis. Deductive and inductive codes were created based on research questions, field notes, and participant responses. A codebook was designed including code definitions, inclusion and exclusion criteria, and examples of code use. Thematic analysis was conducted to identify major themes and supply chain bottlenecks. These were then compared across districts, occupation groups, and supply chain levels (e.g. district, block, health sub-centre, and village). Supply chain bottlenecks were identified throughout the transcripts and compared across groups of respondents to highlight key similarities and differences. Practical recommendations were derived from respondent suggestions, published evaluations of Bihar’s drug supply chain, and analysis of the data. Analyses were conducted using MAXQDA (version 10, VERBI Software).

### Auxiliary nurse midwife surveys

#### Participants

ANMs were chosen as target participants because of their dual role as part of the IFA supply chain and key distributors of IFA. ANMs are responsible for their own drug supply management and as the last to receive IFA, are most affected when both district and block shortages occur.

ANMs predominantly work at health sub-centres, which should serve a population of 3000–5000 [25], and coordinate IFA distribution with ASHAs and AWWs, who work at the village level (Anganwadi Centre Population Norm: 400-800) [26]. In Bihar, actual coverage estimates are much larger with health sub-centres serving almost 10,000 [27] and Anganwadi Centres over 1000 [28, 29].

#### Data collection

We constructed a 37-item survey in collaboration with CARE staff to assess ANM IFA supply and protocols. We randomly selected 3 ANMs from each of the 137 blocks across 8 districts from lists provided. If an ANM was no longer assigned to that block or refused, then another ANM was randomly chosen. CARE block coordinators, trained on survey contents and overall study objectives, administered surveys to ANMs. Names were not collected to ensure confidentiality (see Additional file 9 for ANM questionnaire).

#### Data management and analysis

Trained research assistants input survey data into Excel spreadsheets (Microsoft Excel 2010, Redmond, WA). Quality checks were completed by the primary author.

All analyses were conducted using SAS v9.2 (SAS Inc., Cary, NC, USA). We calculated survey weights to account for non-responses, differing numbers of ANMs per block, and blocks per district. Of the 137 blocks invited to participate, 11 blocks did not respond. Represented blocks (*n* = 126) were compared with non-responsive blocks (*n* = 11) on 7 antenatal characteristics from CARE programme baseline data. We found these 11 blocks had fewer pregnant women receiving FLW counselling during their last pregnancy on pregnancy danger signs (*p* < 0.0001), emergency preparedness (*p* = 0.04), and family planning (p < 0.0001). We found no significant differences comparing the number of mothers who during their last pregnancy: received at least 90 IFA tablets, were visited two or more times during their last trimester by any FLW, received advice from FLWs on immediate new-born care, or delivered their last child at a health facility (data not shown) (Unpublished data, CARE India).

### Ethics

To maintain confidentiality, all districts were assigned letters (A-H) and health workers are referred to as state, district, and block officials. This was done because for some professions, only one post is occupied in each district. ANMs, AWWs, and ASHAs are referred to by their titles and all storekeepers, including pharmacists and clerks, are referred to as storekeepers.

The Institutional Review Board of Emory University approved this study’s protocol, considering it “exempt human subjects research”. Local IRB approval was not required as this research was considered part of CARE’s program operations, which were conducted with the permission of the Government of Bihar. All respondents provided informed oral consent, which was selected as the research presented minimal risk and a signed consent would be the only document linking the participant to the study.

## Results

### Iron and folic acid procurement and distribution

**Fig. 1:**
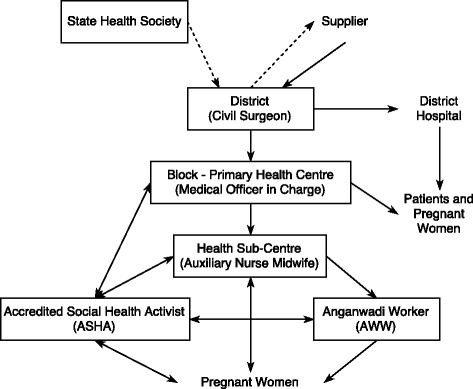
Major components of the IFA supply chain in Bihar, India. Flow chart showing the distribution of iron and folic acid supplements and funds from the state level (State Health Society) to the beneficiaries (pregnant women). **Dotted lines**: funds for purchase of IFA supplements; **Solid lines**: iron and folic acid supplements; **Boxes**: Surveyed populations; **PHC**: Primary Health Centre; **ANM**: Auxiliary Nurse Midwife; **ASHA**: Accredited Social Health Activist; **AWW**: Anganwadi Worker

**Fig. 2:**
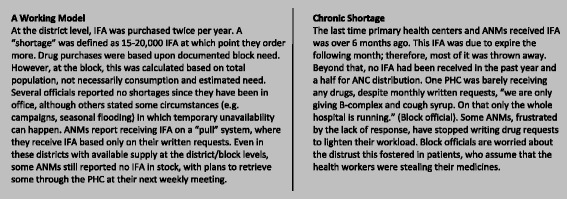
A district comparison. To demonstrate the range of IFA stock and supply chain practices, a brief comparison was made of two districts based on those with a higher and lower functioning IFA supply chain

We present here the intended route of IFA from procurement to beneficiary in Bihar (Fig. 1). It should be noted that IFA procurement is largely similar to other medicines listed on the Essential Drug List [30], which are often purchased and distributed together. To our knowledge, there was no singular document which outlined this entire process. However, we gained an understanding of this pathway through interviews, policy documents, and previous research. Lack of clarity and documentation of this process has been cited as a key barrier to successful medicine distribution in Bihar [31]. This has also led to variability in supply chain performance across districts (Fig. 2).

Procurement begins with selection of companies and fixed rates of drugs by the Bihar State Health Society through a competitive bidding process [31–33]. Bihar government documents state that district funding is allocated based on previous consumption [31, 34]. Actual purchasing is decentralized to the district. The Civil Surgeon submits purchase orders based on State Health Society and District Store information, which is then approved by the District Magistrate. This system changed slightly with the establishment of the Bihar Medical Services and Infrastructure Corporation Limited (BMSICL), which centralized drug purchasing to a large extent. Districts now submit purchase orders to BMSICL, who then procures drugs from suppliers and distributes to regional warehouses [35]. As per policy, all districts are to purchase enough drugs for 6 months with a second order after 3–4 months to allow a 2–3 month buffer supply [34]. Districts are responsible for retrieving drugs from Patna-based depots with payment in hand (Cash and Carry) [34]. Drugs should then be distributed to the blocks according to their estimated need (submitted as a written request or ‘indent’) [34, 36]. Indents from the block store must be approved by the Medical Officer in Charge, the Civil Surgeon, and finally sent to the District Store for fulfilment. From the primary health centre, ANMs receive IFA to distribute at their health sub-centres and monthly Village Health, Sanitation, and Nutrition Days (VHSNDs) [37]. Though IFA distribution and counselling to pregnant and lactating women is clearly outlined [37, 38], there is no clear policy specifically pertaining to health sub-centre drug requests and stock management [34]. At the village level, ASHAs receive IFA independently through ASHA drug kits [39]. AWWs do not receive IFA for antenatal care distribution (Fig. 1). All three are charged with coordinating IFA distribution to pregnant women in their coverage areas through VHSNDs [38]. However, specific roles and stock management between the three positions are not clearly defined.

### ANM survey results

In total, 340 ANM surveys were completed for an 83% response rate. On average, ANMs reported an average coverage population of 9471 (Table 2), approximately twice the government norm. Only 55.9% of ANMs reported having IFA in stock on the day of the survey (Table 3). Those that did reported on average 4306 tablets (Table 2). This varied greatly by district from 0 to 90.5% (Table 3). Few ANMs distributed any IFA to AWWs (21%) or ASHAs (39%) in the last month (Table 2).

**Table 2:** Coverage, iron and folic acid stock, and distribution as reported by ANM, per Health Sub-Centre

Characteristic	n	Mean (95% CI)
Population served by health sub-centre	288	9471 (8848, 10,094)
Pregnant women registered in previous month	313	20 (19, 22)
Current IFA stock (number of tablets)	331	2325 (1744, 2906)
Current IFA stock, if present (number of tablets)	182	4306 (3283, 5329)
Number of IFA tablets given to AWW in previous month, if given	71	1547 (899, 2195)
Number of IFA tablets given to ASHA in previous month, if given	131	619 (357, 881)

Extreme values were excluded from each category

*ANM* Auxiliary Nurse Midwife, *IFA* iron and folic acid, *AWW* Anganwadi Worker, *ASHA* Accredited Social Health Activist

**Table 3:** ANM reported iron and folic acid supplement supply status and procurement protocols by district

	IFA Out of Stock (%)	Cannot Request IFA from PHC (%)^a^	Uses Buffer Stock (%)^b^	Average Buffer Stock (mean (95% CI))^c^	Only obtains IFA from the PHC (%)^d^
District A	90.5	17.3	59.1	447 (265, 628)	81.9
District B	28.1	7.0	87.4	911 (355, 1467)	63.2
District C	63.3	5.3	81.4	544 (219, 867)	64.1
District D	0.0	7.3	92.6	1112 (520, 1704)	81.7
District E	26.0	17.5	69.6	1196 (425, 1967)	82.8
District F	47.8	14.3	60.2	1182 (246, 2118)	78.1
District G	16.2	3.5	82.1	303 (121, 485)	41.0
District H	13.8	19.8	42.6	1177 (613, 1743)	82.7
Total	44.1	9.5	74.4	751 (554, 949)	67.8

*ANM* Auxiliary Nurse Midwife, *IFA* iron and folic acid supplements (100 mg elemental iron and 500 mcg folic acid), *PHC* Primary Health Centre, *CI* Confidence Interval

^a^ANMs responded to a multiple choice question asking how they receive IFA from the PHC with 3 possible responses: through fixed deliveries (cannot request), through fixed deliveries (can request), or can only receive IFA through request

^b^Alternate responses were: request IFA when completely out of stock or cannot request IFA

^c^Average buffer stock was calculated only among those ANMs reporting buffer stock use

^d^Alternate responses were: can take IFA from ASHA kits, can borrow IFA from other health sub-centres, or can purchase IFA using health sub-centre funds; On average, 6.9% of survey data was missing from the above table

Almost 10% of ANMs reported IFA receipt only through fixed deliveries (push-system). Over one-fifth (21.8%) of ANMs said they only request IFA after they are completely out, meaning they do not maintain a buffer stock (Table 3). Those that did keep a buffer supply reported a wide range of levels with a mean estimate of 751 tablets (Table 3). ANMs who did report using a buffer stock system were more likely to have IFA in stock at the time of the survey (OR: 3.0 [1. 5, 6.0] (*p* = 0.0022)). Only 2.9% of ANMs reported that they could use health sub-centre funds to purchase IFA locally (data not shown) while 67.8% said they could only receive IFA through the primary health centre (Table 3).

### Qualitative data results

Our analysis of the in-depth interviews revealed several key bottlenecks at various levels in the IFA supply chain.

#### IFA need

IFA need was most often reported as calculated based on district size with 3–3.3% of the total population estimated to be pregnant women. Lactating women, despite being entitled to IFA by policy mandate [9], were not included in this estimate. Some officials emphasized that data from the blocks were not even needed to arrive at this estimate. *‘No! We don't need a requirement [from the primary health centres] for placing the order. The number of pregnant women... is calculated. So we place [the order] as per the population’* (District F Official).

#### IFA requests

##### IFA purchasing – District

According to the state official, suppliers should have medicines ready within 45 days for the first order and 21 days for subsequent orders. To increase supplier accountability to this timeline, the State Health Society established a penalty for late deliveries. *‘If they don’t deliver on time then from 0.5% per day up to 37%, I’ll deduct their money from the payment’* (State Official). However, in almost all districts, drugs were received an estimated 1–3 months after purchase order submission. *‘There is no supplier who will supply you the order on time. This problem is always there in the supply chain. If we place an order, we get it after 3 months’* (District C Official). *‘It takes about one and a half to two months to get the [IFA] delivery’* (District A Official). Reporting delayed shipments to the State Health Society was only mentioned in 2 districts. In contrast, most districts did not articulate any strategies to encourage timely drug delivery. *‘We just wait for the further supply of the tablets from the company’* (District B Official).

##### Block requests

Although the system for requesting IFA was well-known, most health workers did not perceive indents as effective. In District C, a block official did not submit requests based on need alone but called ahead to ask what was available, leading to a lack of documentation of needed drugs which were already out-of-stock. Dependency on the district store was also emphasized, no alternative sources of IFA were perceived to be available in case of district shortages. A block official in District A articulated this frustration with several drugs which were not being obtained through repeated monthly written requests to the district store.

*What will we do? We are helpless. We send the indents regularly to send us folic acid. All basic medicines which are needed to run the hospital, like Paracetamol, Methergine, etc…. even that is not being supplied from the past one and a half years.* (District A Block Official)

As indents were not perceived as effective in obtaining drugs, some explained that this procedure was only conducted in case of audits.

*Well! There is no result of sending the indent. Whenever they get the IFA they inform us and we get it from them. For our safe side, we send the indent so that no one points out that “you were not having IFA, so what did you do about that?”* (District C Storekeeper)

##### ANM requests

We also noted inconsistencies about how ANMs requested IFA from the primary health centre. Block officials reported that ANMs submitted written requests while many ANMs stated that they received IFA from the store without any documentation. Between these fixed deliveries, some ANMs did mention making written or verbal requests if they were out of stock. When the block did not have IFA, ANMs reported that there was no other means to procure IFA for distribution, *‘If there will be no stock at the PHC [primary health centre] then what can we do?’* (ANM, District G). All ANMs were also responsible for transport of drugs to their health sub-centre and paid for this at their own expense. One block storekeeper noted that ANMs often refused to take medicines even when available because of transportation difficulties or perceived apathy.

*They [ANMs] don’t want to take it… we have to make them. At times, they even make excuses like I have not brought my bag this week when I come next week then I will take it. Then they take the medicines afterwards.* (District F Storekeeper)

#### Buffer stock

Although officials and storekeepers spoke of maintaining buffer stocks, extra stock kept to avoid stock-outs between purchases, we did not observe buffer stocks in these stores (*n* = 3). All district and block storekeepers who had recently ordered IFA had done so only when they were completely out of stock. In multiple stores, there was little or no IFA, and storekeepers mentioned the futility of writing indents except in case of audit. In other stores, we were told buffer stocks were unnecessary. *‘We never felt the need of keeping buffer stock and never faced shortage’* (District F Official). Some storekeeping practices also prevented others from maintaining buffer stocks, *‘I distribute only to those whose stock gets over’* (District F Storekeeper).

#### Expiring & expired medicines

The most common strategy reported to handle expiring drugs (IFA shelf life: 17–23 months) was increased distribution. One storekeeper explained *‘We have the pressure of consuming IFA before it gets expired’* (District C Block Storekeeper). Another block described his attempt at returning about-to-expire drugs to the district.

*Then they instruct us to consume the maximum at your level, as there is no need of return, and even it is not done, and to date has not been returned. I cannot even tell you how these medicines are disposed. We just inform the MOIC [Medical Officer In-Charge] and doctor about the status and ask them to prescribe the medicine.* (District A Storekeeper)

He further explained that during the most recent incident, expiring stock was distributed in *Mahadalit Tolas* (the poorest areas). ANMs also reported that excess IFA goes to waste. *‘But now we usually get about to expire medicine in bulk from the Civil Surgeon. It is not possible for us to distribute 10,000s of tablets in a month’* (ANM, District A).

Stock organization and managing expiration dates was also almost exclusively done either by memory or through the stock register. *‘We first keep this in mind, which medicine is of what type, it’s all mind talk. The medicines which seem to expire first we supply them first, it stays in my mind’* (District F Storekeeper). Only one storekeeper showed us a separate sheet with only expiration dates to quickly find which medicines were about to expire.

#### Storeroom

Several district and block officials expressed concern or concessions made because there was not enough space or no fixed location to store medicines. In District A, medicines were stored in district hospital hallways and vacant rooms. Due to this transient space, the storekeeper made a point to distribute medicines quickly as he did not have room to store them. At the block level this trend continued. At one district, the block official and storekeeper mentioned the lack of racks and storeroom disorder.

Regarding ANMs, many told us they stored their medicines with ASHAs, at Anganwadi Centres, or at home because their health sub-centres were not secure. *‘We store IFA in our bags with ourselves. The Health Sub-Centre is there but it is not functional so we don’t store there’* (ANM, District A). Some ANMs did store drugs at their health sub-centre in a locked cupboard or on a rack.

Two storekeepers spoke of rat protection (traps or blocking cracks in the walls) but only one acknowledged the fact that damaged stocks happen.

*How much we can avoid the stock from getting spoiled? Rats enter the store and that is the fact. Sometime the medicines even become damaged in transporting or moving it from one store to another store. However much we try, we won’t be able to save it.* (District G Storekeeper)

#### Personnel & training

##### District/Block

Most health officials did not perceive supply management trainings as helpful.

*No! [Trainings are] not given. See, what happens madam is that I don’t feel that these kinds of trainings are brain teasing… Once they are in the store they continue with their work. It’s not a tough job. The chart is there, you have to fill the serial number and name of the medicine… So filling up these columns is very simple.* (District C Block Official)

However, at many stores we visited, pharmacists were not even available.

*No [I] never received any training, I am a clerk over here, as there is no trained storekeeper or pharmacist. This post is for a pharmacist… but since the government has not arranged for this, still work had to be done so I am doing this.* (District A Storekeeper)

Almost all storekeepers said they did not receive supply management training. Only one district hospital official said he received training from the Indian Institute of Health Management Research through the State Health Society. It is possible that this training may have been specific to hospital issues. Both block and district storekeepers explained that their training came from asking others, not from formal supply management training.

*‘No nothing like that, I have not got any kind of training I just ask from the older people here that what is the requirement.’* (District A Block Storekeeper)

*‘No. I am doing this myself and following the same culture of the person who was working here before me.’* (District H Storekeeper)

#### Health sub-centre/village

According to training materials, FLWs should be conducting coordinated efforts to identify and register pregnant women, bring them to VHSNDs, distribute IFA, and counsel on IFA consumption and benefits [40]. However interviews revealed varying roles across districts for each FLW. This may be due to inconsistent training across districts concerning IFA distribution. ANMs usually stated that all FLWs distributed IFA. However, it appeared that distribution was often conducted by the ANM and one other FLW. In District H, AWWs were responsible for IFA distribution to beneficiaries and to the ASHAs, *‘ANM gives IFA tablets to me… I give [the ASHA] the tablets.’* (AWW, District H). ASHAs here did not take a major role in giving IFA, *‘I don’t distribute much. I write the names of the pregnant women who come to the Anganwadi Centre to take the tablets’* (ASHA, District H). In contrast, an ASHA in District D explained that AWWs did not distribute IFA, *‘No! Apart from the ANM, only ASHA gives [IFA].’* However, in other districts, lack of training was a barrier to ASHA IFA distribution. *‘ASHAs do not give IFA to pregnant women as they are not confident. So they give the IFA to the ANMs to distribute. The ASHA only brings women to the centre. She does the counselling’* (ANM, District A). In another district, ASHAs only distributed under ANM supervision, *‘She brings [her IFA] to the centre. They have not been trained properly… When they come to us with their kit, we explain them which medicine to give and to whom’* (ANM District D).

#### Lack of personnel

The state official mentioned a lack of manpower for the bidding processes in addition to shortages of doctors, pharmacists, and nurses. The lack of Bihar nursing colleges and teachers for ANM training programs were also mentioned as factors. ‘*There is an acute shortage of doctors and nurses… Here at our ANM schools, there is a shortage of teachers. There are 109 vacancies. For that, only 54 people turned up out of which 34 were placed’* (State Official).

## Discussion

IFA supply is a public health issue in Bihar, the extent of which varies greatly across districts. We discovered specific bottlenecks which impacted IFA forecasting, procurement, expired drugs, storage, and an overall lack of personnel. In addition, few training opportunities existed for important players in the supply chain.

In a 2006 comparative analysis, Bossert et al. found that higher performance logistics systems had decentralized planning and budgeting. Conversely, centralization of information systems and inventory control was associated with greater success [41]. The typical scenarios we found in Bihar were in contrast to this ideal scenario. Forecasting future IFA need was usually done using a top-down approach (e.g. calculations from population estimates) rather than based on actual needs. Adjusting orders to fluctuating demand was not possible in most cases because when IFA was unavailable, primary health centre and ANM requests were often not documented. Annual purchasing instead of the recommended 3-4 month timelines also inhibited dynamic forecasting. Though Bihar’s inappropriate forecasting mechanisms have been cited as problematic in previous research and reports [42, 43], this has usually been seen as a state and district level issue. Our data highlights these issues at the block and health sub-centre level as well. Therefore, to fully address this, changes must also occur at these lower levels to accurately track and react to changing population needs. Similarly, we also found a need for documentation standardization and inventory control. The Bihar government has recognized this need and began implementation to computerize stock information at the district and block levels through BMSICL [34, 44]. However, this has not been fully rolled out and remains an outstanding issue in current government progress reports [23]. To these strategies, we also recommend increased documentation of sub-centre and village levels to more accurately track existing inventories and needs (Table 4).

**Table 4:** Identified bottlenecks of Bihar’s IFA supply chain, proposed actions, and key stakeholders

Identified bottleneck	Proposed action	Key stakeholders
Lack of appropriate IFA need forecasting	Standardized demand forecasting based on accurate estimates of district needs and previous consumption	SHS, BMSICL, District Officials^a^, District Storekeeper, Block Officials^b^, Block Storekeeper, ANM
	Computerization and clear documentation of inventory, stock requests, and expiry dates	SHS, BMSICL, District Officials^a^, District Storekeeper, Block Officials^b^, Block Storekeeper, ANM
	Estimates to include lactating women population; IFA distribution and counseling standard	SHS, BMSICL, District Storekeeper, Block Storekeeper, ANM, ASHA, AWW
Late supplier deliveries resulting in inconsistent supply	Utilization of updated BMSICL policy to deduct payment upon late delivery, damaged stock, etc.^c^	SHS, BMSICL, District Officials^a^, District Storekeeper
Indents not being utilized nor perceived as effective	Training and monitoring to assure indent use and effectiveness	SHS, BMSICL, District Officials^a^, District Storekeeper, Block Officials^b^, Block Storekeeper, ANM, ASHA
Perceived or actual inability to procure IFA when needed through local purchasing	Explore use of untied funds through *Rogi Kalyan Samiti* or others to purchase IFA locally in times of shortage	SHS, BMSICL, District Officials^a^, District Storekeeper, RKS, Block Officials^b^, Block Storekeeper, ANM, ASHA
Lack of buffer stock use at all levels	Implementation, monitoring, and evaluation of existing buffer stock requirements^d^	SHS, BMSICL, District Officials^a^, District Storekeeper, Block Officials^b^, Block Storekeeper, ANM, ASHA
	Ensure adequate storage facilities so stock can be stored safely	SHS, BMSICL, District Officials^a^, District Storekeeper, Block Officials^b^, Block Storekeeper, ANM
No safe disposal plan for expired medicines and pushing of expiring drugs to patients and frontline workers	Transparent plan to prevent expired medicines through appropriate purchasing practices and safe disposal of expired medicines	SHS, BMSICL, District Officials^a^, District Storekeeper, Block Officials^b^, Block Storekeeper, ANM, ASHA
Storeroom transiency and disorder	Construct, purchase, or long-term rental of adequate storerooms. Funding for racks, labels, and shelves.	SHS, BMSICL, District Officials^a^, District Storekeeper, Block Officials^b^, Block Storekeeper, ANM
	Training for storekeepers including storeroom order and inventory protocols.	SHS, BMSICL, District Officials^a^, District Storekeeper, Block Officials^b^, Block Storekeeper
Inconsistent training on IFA counseling/distribution across FLW types	IFA counseling/distribution training for all frontline workers who work with pregnant women	Block Officials^b^, ANM, ASHA, AWW
	Training for all frontline workers together at health sub-centre level to improve coordination and communication	Block Officials^b^, ANM, ASHA, AWW

*SHS* State Health Society, *BMSICL* Bihar Medical Services and Infrastructure Corporation Ltd., *ANM* Auxiliary Nurse Midwife, *ASHA* Accredited Social Health Activist, *AWW Anganwadi* Worker, *RKS Rogi Kalyan Samiti* (Patient Welfare Committee)

^a^District Officials, Civil Surgeon, District Programme Manager

^b^Block Officials, Medical Officer In Charge, Block Health Manager

^c^Bihar Medical Services and Infrastructure Corporation Ltd. Bid document for supply of drugs & medicines for various medical institutions of Government of Bihar for the year 2013–14. Patna, Bihar: BMSICL; 2013: http://bmsicl.gov.in/uploads/Drug%20Tender%20new.pdf. Accessed 05/11/2014

^d^State Health Society, National Rural Health Mission, Government of Bihar. *Consolidated Revised NRHM State Project Implementation Plan 2012–13 of Bihar.* Patna, Bihar, India2011

Improved inventory documentation when fully implemented should bring to light another bottleneck that we found, late supplier deliveries. For this, the obvious strategy is to enforce existing policy as the State Health Society already has penalties in place [44]. Timely reporting is needed from the districts which could be improved through improved communication and process monitoring. This process may have changed substantially as purchasing and delivery are now done directly through BMSICL [44], however reporting of late deliveries remains a potential issue which could affect drug supplies.

As previously discussed, an integral component of successful forecasting is an accurate picture of need. However, we found that many IFA requests were either not being made or recorded. This was due often to convenience or perceptions that these requests were not effective. This occurred particularly during stock-outs, which has been recorded previously [42, 45]. Training to emphasize the importance of documenting need, especially during times of shortage, as well as an improved response to these requests are both critical to improve this vital part of the supply chain especially at more peripheral levels (Table 4). During prolonged stock-outs, health facilities could implement the use of *Rogi Kalyan Samiti* (untied) funds for local IFA purchases (Table 4). Use of these funds however, requires a functioning Village Health Nutrition and Sanitation Committee, cooperation between the health sector and panchayat leaders, and awareness of how to spend funds, which have been reported as barriers to fund use in Uttar Pradesh and Maharashtra [46, 47].

We also found that district and block stores were not preparing for stock-outs or late supplier deliveries as evidenced by an overall lack of buffer stocks. Though many storekeepers mentioned buffer stocks and knew their purpose, most did not re-order until completely out of stock. Enforcing current government policy and incorporation of computerized reporting should facilitate appropriate ordering practices, which has been shown to ensure a more consistent drug supply [41] (Table 4).

In contrast, many districts were also experiencing surpluses of expired or about-to-expire IFA, sometimes in the same districts where stock-outs occurred. This seemed to happen in part because of the poor request practices mentioned previously. This often led to large influxes of about-to-expire IFA to FLWs and providers with instructions to increase distribution. Improving responses to IFA requests and documentation should mitigate some of this excess. When medicines do expire, transparent plans should be in place for proper disposal at the block or district level to avoid improper disposal at the village level. Use of standard inventory practices such as FEFO (first to expire, first out) should also be enforced to prevent these excesses [48].

The implementation of many of these changes will require increased capacity of both storerooms and storekeepers. Currently, many storerooms are in temporary locations, which often restricted the amount of stock that could be stored and investments in shelving or security (Table 4). Furthermore, although storekeepers overall were receptive to supply management trainings, many officials viewed the storekeeper role as simple and did not see value in further preparation. FLWs also assume responsibility for managing their own IFA stock and communicating their need to the block stores. FLW training on supply management could help to teach basic inventory knowledge (e.g. buffer stocks) and emphasize the use of written requests, to enable accurate documentation of need. Holding trainings through the health sub-centre platform would also allow for coordination and communication between the FLWs, as this would bring together ANMs, ASHAs, and AWWs within a single health sub-centre coverage area [24]. This training should also include IFA distribution to lactating women, as the majority of FLWs we interviewed did not distribute IFA to this population (Table 4).

This study has several strengths. We interviewed a wide range of people at different levels in Bihar’s IFA supply chain. Also, in each district we interviewed at least one representative from each level of the supply chain. Through ANM surveys, we also supported our qualitative work by quantitatively demonstrating the variable IFA supply and associated inventory control practices. By utilizing a mixed methods approach, we were not only able to identify bottlenecks along the supply chain but also explore the reasons and justifications for not following certain policies, which would not be possible by conducting quantitative surveys alone.

There were also some limitations to this research. This study is largely qualitative and therefore is not generalizable. However, the 8 districts included were originally selected by CARE as focus districts in order to reflect the state’s geographical and political diversity. In addition, we did find that many identified themes agreed with existing supply chain literature from India and other countries. Therefore, bottlenecks identified here may be useful to assess in similar contexts when examining supply chain issues. Though we did talk to several groups involved in IFA receipt and distribution, we did not interview suppliers, drug inspectors, or district magistrates, who also play significant roles in the IFA supply chain. These individuals may provide additional insight into strengths and weaknesses of the current system. Finally, our chosen methodologies were not optimal for detecting the influences of corruption in Bihar’s IFA supply chain. Incidences of corruption have been reported in India’s health system [46, 49] and in 2014, several officials in BMSICL, which was established to improve drug procurement, were accused of mismanagement in medicine and equipment purchasing. The resulting legal actions halted centralized procurement, causing widespread stock-outs in the state [22, 50, 51]. Further research will be required to understand the impact of corruption on the Bihar supply chain and how good governance practices, such as World Health Organization recommendations [52], can be incorporated into the existing framework.

Previous evaluations of Bihar’s essential medicine distribution have also highlighted several bottlenecks identified through this analysis [31, 43, 53]. The creation of the BMSICL [44], was envisioned to address these by streamlining state-level drug procurement and improving documentation protocols. This followed a pattern initiated by several Indian states to emulate Tamil Nadu’s drug procurement model, recognized as highly successful [49, 54]. Such strategies like centralized tendering and purchasing, regional warehouses, and computerized inventory management were planned in order to reduce costs and increase essential medicine accessibility [44, 55]. In coordination with local organizations, BMSICL has been successful in some of these goals such as warehouse construction, however widespread stock-outs are still being reported [22, 44]. In addition, Bihar has increased IFA purchases to incorporate distribution to lactating women [34], however, according to our data FLWs still do not distribute to this group regularly.

Although the Bihar government is taking steps forward, many of the issues raised in our study may not be resolved by the changes underway. The drug procurement changes focus primarily on the drug tendering process, regional warehouse construction, and improved demand assessment. However, the poor practices we discovered at the district, block, health sub-centre, and village must be addressed to attain a fully-functioning drug supply system. This will become increasingly important in light of the ‘Iron+ Initiative,’ which expands universal IFA supplementation to children from 0 to 19 years and women of reproductive age in addition to pregnant and lactating women. This programme also introduces additional types of IFA tablets, which vary in iron content, size, and color [56]. Especially with the implementation of this programme and existing demands, improving the IFA supply chain will be critical to this initiative’s success.

In many iron supplementation programs, low IFA consumption has been assumed to be due to poor adherence, and often attributed to individual factors. However, when the supply chain itself is dysfunctional, this too reduces the value of the intervention in the eyes of the beneficiaries. Inconsistent supply can foster distrust among patients for the healthcare system as a whole [57]. Strengthening the Bihar supply chain will be critical to improving iron supplementation programs in Bihar. More research will be useful to examine how effective monitoring and evaluation can be achieved in addition to the feasibility and maintenance of a more dynamic, flexible supply chain system.

## Conclusion

In this assessment of Bihar’s IFA supply, we found that both supply availability and management practices varied depending on the district, especially at the block and village levels. Specifically, we discovered issues regarding IFA forecasting, procurement, expired drugs, storage, and an overall lack of personnel. Training on these topics were also often perceived to be unnecessary, and therefore few opportunities existed to change these management practices. Changes are being made at the top levels through reorganization of how stocks are purchased and other measures. But improved practices at the block and village level and improved responsiveness from the state and district will be necessary to truly improve the IFA supply chain in Bihar.

## Additional files

Additional file 1: IDI State official: In-depth interview guide for state level official. (DOCX 20 kb)

Additional file 2: IDI District official: In-depth interview guide for district officials. (DOCX 19 kb)

Additional file 3: IDI District storekeeper: In-depth interview guide for district storekeepers. (DOCX 21 kb)

Additional file 4: IDI Block official: In-depth interview guide for block officials. (DOCX 19 kb)

Additional file 5: IDI Block storekeeper: In-depth interview guide for block storekeepers. (DOCX 19 kb)

Additional file 6: IDI ANM: In-depth interview guide for Auxiliary Nurse Midwives. (DOCX 20 kb)

Additional file 7: IDI ASHA: In-depth interview guide for Accredited Social Health Activists. (DOCX 18 kb)

Additional file 8: IDI AWW: In-depth interview guide for Anganwadi workers. (DOCX 18 kb)

Additional file 9: Questionnaire ANM: Survey administered to Auxiliary Nurse Midwives. (DOCX 18 kb)

## Abbreviations

ANM: Auxiliary Nurse Midwife
ASHA: Accredited Social Health Activist
AWW: Anganwadi Worker
FLW: frontline worker
HSC: health sub-centre
ICDS: Integrated Child Development Scheme
IFA: iron and folic acid
NRHM: National Rural Health Mission
SHS: State Health Society
VHSND: Village Health, Sanitation, and Nutrition Day
