# Supplementary material for: Identifying bottlenecks in the iron and folic acid supply chain in Bihar, India: a mixed-methods study
Source: BMC Health Serv Res. 2018 Apr 12;18:281. doi: 10.1186/s12913-018-3017-x (PMC5898001; doi:10.1186/s12913-018-3017-x)
Supplement: Supplementary file 1 — IDI State official: In-depth interview guide for state level official. (DOCX 20 kb) [file 12913_2018_3017_MOESM1_ESM.docx]

*Due to the iterative and reflexive nature of qualitative research, this document served to guide the interviews with the participants and was not followed word for word. In some cases, questions may have been skipped, asked in a different order, or other questions added according to the participants’ responses and flow of the conversation.*

**IFA SUPPLY INTERVIEWS – State Level**

**START TIME OF INTERVIEW _______:________ AM / PM**

**INTRODUCTION**

1. Could you describe what you see as ***your* role** in the procurement or distribution of IFA?
   1. What processes are you involved in?
   2. *Monitoring? Evaluating companies? IFA distribution protocols?*
2. Could you walk us through the process of how drug companies (for IFA) are chosen as **government approved companies**?
   1. How are **quality** of products and **price** assessed? How often is quality assessed? (per batch? Year? X years?)
   2. Who are the major players in this process?
   3. How frequently are tenders / companies evaluated?
   4. Is this process different for **ASHA kits**? Are the funds available for ASHA kits different than those used for other IFA sources? (100mg, 20mg, syrup) How about the funds available for ASHA kit replenishment (eg. When she runs out of IFA)?
3. Under which **heads** are IFA funds covered? Is there a head specific for IFA? Is it the head of MCH or RCH? Drug procurement? Is there a different head for ASHA kits?
   1. How are funds distributed across to the districts?
   2. Are there times when drugs are directly distributed to the districts (centralized purchasing)?
4. How are **budgets allocated** to each district for IFA purchase?
   1. Population estimates / what figures
   2. What target groups are included? (PW, adolescents, children (ages), LW)
   3. Do you find that districts purchase according to their estimates (population based) or based on indents received?
5. How much of the district drug procurement budget can be used for **local IFA purchasing**? (%)
   1. Do blocks / PHCs have the option to procure IFA locally?
   2. What is the rationale for this?
6. How are **delivery timelines / quality standards** enforced? Is it up to the district to enforce these or does the state play a part in monitoring company performance after approval?
   1. Describe the state’s role.
   2. Describe the district’s role. What can the district do if a company is late in getting a delivery ready? (% deduction in price? Other action?)
   3. Is there a process for blacklisting a company? Has any company ever been blacklisted? (Could you describe what they did to become blacklisted?)
   4. What is the contingency plan if this happens and drug distribution is halted to the districts?
   5. Is there a **contingency stock** or emergency stock held by the state in case there is a major drug distribution problem for any reason?
7. Are **quality checks** of IFA done at a state level as well as district level?
   1. What is this process?
8. I read that there is a **Cash and Carry method** for all drug procurement, including IFA. Why was this chosen?
   1. What funds are available to the districts to pay for drug transport?
   2. What **vehicles** are available to the districts to transport drugs?
   3. Are there any guidelines for how districts should be receiving drugs in the case that they don’t have a specific vehicle allocated for drug transport?
   4. Are there any districts you are aware of in which the company is delivering to them directly? (*1-2 districts had described this)*
   5. Where are the drug depots currently (all in Patna? Local warehouses?)
   6. Why don’t company contracts from the state ensure delivery to the district?
9. Once the drugs are received by the district, are there any standardized protocols that should be followed?
   1. At the block level?
   2. Sub-center level?
10. How is IFA distributed between the health department and **ICDS**?
    1. Is this decided in districts only? Or is there a state guideline (for CDPO or AWW distribution of IFA)?
11. I have heard that a new company “**Bihar Medical Services** and Infrastructure Corporation Ltd.” has started and was scheduled to be functional in April. What is the current status of this project?
    1. How will the current situation change in terms of drug procurement?
    2. In terms of drug distribution to the districts?
    3. Quality?
    4. Will cash and carry method still be implemented?
12. How are districts **monitored** by the state for their distribution of drugs?
    1. What paperwork is examined?
    2. Who looks over the district registers?
    3. What are they looking for?
    4. If there was a district that had not been purchasing IFA for some time, are there any procedures in place to detect when this is happening? (e.g. If a ‘utilization contract’ had not been turned in so the district did not have access to the next year’s funds.)
13. How are storekeepers and pharmacists currently **trained** in logistics and supply management? Are there any government trainings provided?
    1. Initial training?
    2. Refresher training?
    3. Or are they supposed to be trained in this before they are hired?
14. We read about some trainings being offered in logistics and supply through the **NIHFW** (National Institute of Health and Family Welfare). Are these types of trainings available to district and block level workers?
    1. Do ANMs receive any training (as they have to manage their stock as well as patients)?
15. What parts of the drug procurement process are standardized across states? Do other states do this process differently? (Any examples?)
16. What policies or guidelines are followed for procurement of drugs at the state level? Are there any separate policies about IFA procurement and distribution? **Could we see or get a hard or soft copy of these?**
    1. Policies of procurement / distribution at the district level?
    2. Distribution of drugs from district down?
    3. Separate policies for IFA given that they are distributed differently from other medications (eg. Not by prescription) and with a different purpose (prevention).
17. Are there any protocols to dictate how this distribution is being monitored? (Eg. What should the MOIC be looking for when he is overseeing the ANM registers? The BCM for the ASHA?) **Could we see or get a hard or soft copy of these?**
    1. What are they?
18. Would it be possible to see the **IFA purchase orders** that have been approved for our 8 districts of focus for the past 3 years? (100mg, 20mg, syrup; ASHA kits)
    1. Who would have that information?
19. Do you have any questions for us?
    1. Do you have any additional comments that you think we should know?
    2. Is there anyone you would recommend us talking to in order to receive additional information on the IFA supply and distribution here?

NAMES & CONTACT INFO: ____________________________________________________________________________________________________________________________________________________________________________________

Thank you so much for your time and participation today. It has helped is greatly in understanding the Iron and folic acid supplementation supply chain here in Bihar state. If we have further questions or inquiries about the IFA supply, would it be alright to contact you again?

**END TIME OF INTERVIEW _______:________ AM / PM**
