# Supplementary material for: Identifying bottlenecks in the iron and folic acid supply chain in Bihar, India: a mixed-methods study
Source: BMC Health Serv Res. 2018 Apr 12;18:281. doi: 10.1186/s12913-018-3017-x (PMC5898001; doi:10.1186/s12913-018-3017-x)
Supplement: Supplementary file 2 — IDI District official: In-depth interview guide for district officials. (DOCX 19 kb) [file 12913_2018_3017_MOESM2_ESM.docx]

*Due to the iterative and reflexive nature of qualitative research, this document served to guide the interviews with the participants and was not followed word for word. In some cases, questions may have been skipped, asked in a different order, or other questions added according to the participants’ responses and flow of the conversation.*

**IFA SUPPLY INTERVIEWS**

**_________ DISTRICT ______________ DESIGNATION**

**START TIME OF INTERVIEW _______:________ AM / PM**

**INTRODUCTION**

1. Could you walk us through the process of where your IFA supply comes from, how it comes here, and how it is sent from the district?
   1. How does this change if there are delays in shipments? Insufficient supply?
   2. *How is this process different for 20 mg IFA tablets?*
   3. *How is this process different for ASHA kits? Are the funds available for ASHA kits the same?*
2. **What is YOUR role in the receipt of IFA? What do you submit and approve to request IFA for your district?**
3. Now we’d like to understand the process of how you receive iron and folic acid tablets.
   1. *Who* delivers the IFA to your district?
      1. (A company, which one? Government? Always same person/group?)
      2. How is it delivered? Delivery truck from company? Have to hire a truck?
      3. **Where is it delivered from? Factory in Patna? Local warehouse? Delhi?**
   2. How often are they delivered?
      1. What frequency?
      2. When they were *last* delivered?
      3. How much was received in the *last* delivery?
   3. How do you request deliveries? Please describe the process.
      1. Forms involved? **What does each form look like? (PHOTO OR COPY)**
      2. **Who is contacted?**
      3. What happens when you run out of IFA tablets before the next scheduled delivery?
         1. Same request process? Different? Is there one?
         2. Please describe this process if different.
            1. Different forms?
            2. Different people to contact?
   4. How are quality and quantity of shipments verified?
      1. **Is there a check-list of how quality is verified for IFA that we could see? (Take photo if possible)**
   5. Where are shipments of IFA stored?
      1. District hospital? Other location? **Can we see this place? (Is it close?)**
   6. About how much time passes from IFA receipt to distribution to the blocks (PHCs)?
      1. Is distribution done at scheduled intervals? What are they?
      2. Connected with distribution of other goods/medications?
   7. **How is this process monitored? Are there any audits or evaluations done of this process?**
      1. **Who does them? How often?**
   8. **Are there trainings offered about logistics and supply managing?**
      1. **Who offers them?**
      2. **Who are they offered for? How often?**
      3. **What are reasons people do not attend the trainings? Cost? Travel/time?**
   9. **Is this how it works in ALL districts? Or do other districts do it differently that you know of?**
4. From here, how is IFA sent to the primary health centers?
   1. Who delivers the IFA to the primary health centers?
   2. How often are they distributed?
      1. What frequency? (eg. every month?)
      2. When were they *last* distributed?
      3. How much was last distributed to block level destinations? (total if possible, or a few example blocks)
   3. How do blocks (PHCs) request IFA from the district?
      1. **Forms involved?**
      2. **Who submits these forms?**
      3. **Who do they submit the forms to?**
      4. How do **PHCs** request more IFA if they run out before the next scheduled delivery?
   4. **How is this process monitored? Are there any audits or evaluations done of this process?**
      1. **Who does them? How often?**
   5. **How is the IFA distributed between the health department and ICDS? [to the MO/IC, CDPO]?**
      1. *Whose decision is this?*
      2. Is there a policy in place which defines IFA distribution between the ICDS and health department?
   6. When was the last time you were unable to deliver IFA supplements on time?
   7. Can you describe to me what happened?
5. We’d also like to learn a little bit about how you decide how many iron and folic acid supplements are **needed** in your district.

*IFA Need*

- 1. What is the process that you go through to identify how many IFA supplements you need to request in your district?
     1. What data do you use to come to these estimates?
     2. **Do you estimate number of lactating women for these estimates? Adolescents? Children? How?**
     3. What was the number of IFA supplements (100mg) that you estimated a need for last year? ________________________________
     4. What was the number of IFA supplements (100mg) that you requested from the district last year? _____________________________

*IFA Distribution by FLWs*

- 1. Which frontline workers in your district administer IFA to pregnant women?

1. **What POLICIES or GUIDELINES do you follow during receipt and distribution of drugs?**
   1. **What are they called?**
   2. **Could we SEE a copy?**
2. Would it be possible to see the iron and folic supplements that have not yet been distributed?
   1. [*Verify 100mg tabs and 20mg*] __________________
   2. [*Check expiration date*] __________________
   3. [*Note conditions of storage: climate controlled? Dry? How many supplements are there?*] __________________
   4. **Could we see your stock inventory / registration documents? __________________**
3. Do you have any questions for us?
   1. Do you have any additional comments that you think we should know?
   2. **Is there anyone you would recommend us talking to in order to receive additional information on the IFA supply and distribution here?**

NAMES & CONTACT INFO: ____________________________________________________________________________________________________________________________________________________________________________________

Thank you so much for your time and participation today. It has helped is greatly in understanding the Iron and folic acid supplementation supply chain here in Bihar state. If we have further questions or inquiries about the IFA supply, would it be alright to contact you again?

**END TIME OF INTERVIEW _______:________ AM / PM**
