# Supplementary material for: Identifying bottlenecks in the iron and folic acid supply chain in Bihar, India: a mixed-methods study
Source: BMC Health Serv Res. 2018 Apr 12;18:281. doi: 10.1186/s12913-018-3017-x (PMC5898001; doi:10.1186/s12913-018-3017-x)
Supplement: Supplementary file 3 — IDI District storekeeper: In-depth interview guide for district storekeepers. (DOCX 21 kb) [file 12913_2018_3017_MOESM3_ESM.docx]

*Due to the iterative and reflexive nature of qualitative research, this document served to guide the interviews with the participants and was not followed word for word. In some cases, questions may have been skipped, asked in a different order, or other questions added according to the participants’ responses and flow of the conversation.*

**IFA SUPPLY INTERVIEWS**

**_________ District Storekeeper**

**START TIME OF INTERVIEW _______:________ AM / PM**

**INTRODUCTION**

1. Could you walk is through the process of where your IFA supply comes from, how it comes here, and how it is sent from the district?
   1. How does this change if there are delays in shipments? Insufficient supply?
   2. *How is this process different for 20 mg IFA tablets?*
   3. *How is this process different for ASHA kits?*
2. Now we’d like to understand the process of how you receive iron and folic acid tablets.
   1. *Who* delivers the IFA to your district?
      1. (A company, which one? Government? Always same person/group?)
      2. How is it delivered? Delivery truck from company? Have to hire a truck?
      3. **Where is it delivered from? Factory in Patna? Local warehouse? Delhi?**
   2. How often are they delivered?
      1. What frequency?
      2. When they were *last* delivered?
      3. How much was received in the *last* delivery?
   3. **How is quantity and quality verified?**
      1. **Who does this?**
   4. How do you request deliveries? Please describe the process.
      1. **Forms involved**? **What does each form look like? (PHOTO OR COPY)**
      2. **Who is contacted**?
      3. What happens when you run out of IFA tablets before the next scheduled delivery?
         1. Same request process? Different? Is there one?
         2. Please describe this process if different.
            1. Different forms?
            2. Different people to contact?
   5. Where are shipments of IFA stored?
      1. District hospital? Other location? **Can we see this place? (Is it close?)**
      2. **How do you organize your stocks? Any methods to keep track of expiry dates, order of drugs?**
      3. **How often do you do inventory of your stores? Can you describe that process to me?**
      4. **How do you identify expired stock?**
      5. **Where do you put your expired drugs?**
      6. **How do you make sure your stock does not get damaged while in storage?**
   6. **How is this process monitored? Are there any audits or evaluations done of this process?**
      1. **Who does them? How often?**
   7. **How were you trained logistics and supply managing?**
      1. **At beginning of this job? Were there additional trainings?**
      2. **Who gives the trainings? How often?**
      3. **Are they mandatory? Optional?**
      4. **Do you always attend these? If not, why?**
   8. About how much time passes from receiving IFA until it is sent out to the Primary Health Centers?
      1. Is distribution done at scheduled intervals? What are they?
      2. Connected with distribution of other goods/medications?
   9. **Is this how it works in ALL districts? Or do other districts do it differently that you know of?**
3. From here, how is IFA sent to the primary health centers?
   1. Who delivers the IFA to the primary health centers?
   2. Where are they dropped off?
      1. PHCs? Other locations (Warehouse?)
   3. How often are they distributed?
      1. What frequency? (eg. every month?)
      2. When were they *last* distributed?
      3. How much was last distributed to block level destinations? (TAKE A PICTURE OF THIS PAGE IN THE STOCK REGISTER OR hand copy a few example block distribution dates and totals)
   4. How do blocks (PHCs) request IFA from the district?
      1. **Forms involved?**
      2. **Who submits these forms?**
      3. **Who do they submit the forms to?**
      4. How do PHCs request more IFA if they run out before the next scheduled delivery?
   5. **How is this process monitored? Are there any audits or evaluations done of this process?**
      1. **Who does them? How often?**
   6. **How is the IFA distributed between the health department and ICDS? [to the MO/IC, CDPO]?**
      1. *Whose decision is this?*
      2. Is there a policy in place which defines IFA distribution between the ICDS and health department?
   7. When was the *last* time IFA supplements were not distributed before PHCs ran out of supply?
      1. Can you describe to me what happened?
   8. **Do all districts distribute to their blocks like this? Do they do it differently that you know of?**
   9. **Thinking of ALL the forms and procedures you just described which of these are on a computer registry? Which are done BY HAND?**
4. We’d also like to learn a little bit about how you decide how many iron and folic acid supplements are **needed** in your district.

*IFA Need*

- 1. What is the process that you go through to identify how many IFA supplements you need to request in your district?
     1. What data do you use to come to these estimates?
  2. Do you have or know where we could obtain these numbers?
     1. Number of IFA (100mg) supplements needed in  *your* district (estimate for last year) ______________
     2. Number of different pregnant women registered in ANC in district (last year or last month) ________________
     3. Number of IFA supplements or packs administered in the last year (or month) in the district. _________________
     4. Number of IFA supplements or packs delivered to each block in the past year (or month) in the district._________________

1. **What POLICIES or GUIDELINES do you follow during receipt and distribution of drugs?**
   1. **What are they called?**
   2. **Could we SEE a copy?**
2. Would it be possible to see the iron and folic supplements that have not yet been distributed?
   1. [*Verify 100mg tabs and 20mg*] __________________
   2. [*Check expiration date*] __________________
   3. [*Note conditions of storage: climate controlled? Dry? How many are there?*] __________________
   4. **Could we see your stock inventory / registration documents? __________________**
3. **May we see the IFA receipt and distribution data from the last 2 years? Is this available?**
   1. ***______________________ IFA tablets requested: DATE ______________***
   2. ***_______________________ IFA tablets received: DATE ______________***
   3. ***_______________________ IFA tablets distributed to blocks: DATE ________***
   4. ***_______________________ IFA tablets to DH store: DATE________________***
4. Do you have any questions for us?
   1. Do you have any additional comments that you think we should know?
   2. **Is there anyone you would recommend us talking to in order to receive additional information on the IFA supply and distribution here?**

NAMES & CONTACT INFO: ____________________________________________________________________________________________________________________________________________________________________________________

Thank you so much for your time and participation today. It has helped is greatly in understanding the Iron and folic acid supplementation supply chain here in Bihar state. If we have further questions or inquiries about the IFA supply, would it be alright to contact you again?

**END TIME OF INTERVIEW _______:________ AM / PM**
