# Supplementary material for: Identifying bottlenecks in the iron and folic acid supply chain in Bihar, India: a mixed-methods study
Source: BMC Health Serv Res. 2018 Apr 12;18:281. doi: 10.1186/s12913-018-3017-x (PMC5898001; doi:10.1186/s12913-018-3017-x)
Supplement: Supplementary file 5 — IDI Block storekeeper: In-depth interview guide for block storekeepers. (DOCX 19 kb) [file 12913_2018_3017_MOESM5_ESM.docx]

*Due to the iterative and reflexive nature of qualitative research, this document served to guide the interviews with the participants and was not followed word for word. In some cases, questions may have been skipped, asked in a different order, or other questions added according to the participants’ responses and flow of the conversation.*

**IFA SUPPLY INTERVIEWS**

**_________ Block Storekeeper**

**START TIME OF INTERVIEW _______:________ AM / PM**

**INTRODUCTION**

1. Could you walk is through the process of where your IFA supply comes from, how it comes here, and how it is sent from the block?
   1. How does this change if there are delays in shipments? Insufficient supply?
   2. *How is this process different for 20 mg IFA tablets?*
   3. *How is this process different for ASHA kits?*
2. Now we’d like to understand the process of how you receive iron and folic acid tablets.
   1. Who brings the IFA to your block?
      1. (A company, which one? Government? Always same person/group?)
      2. How is it delivered? Delivery truck from company? Have to hire a truck?
   2. How often does IFA come?
      1. What frequency?
      2. When they were *last* delivered?
      3. How much was received in the *last* delivery?
   3. How do you request IFA? Please describe the process.
      1. Forms involved? **What does each form look like? (PHOTO OR COPY)**
      2. **Who is contacted?**
      3. What happens when you run out of IFA tablets before the next scheduled delivery?
         1. Same request process? Different? Is there one?
         2. Please describe this process if different.
            1. Different forms?
            2. Different people to contact?
   4. Where are deliveries of IFA stored?
      1. Primary Health Center? Other location? **Can we see this place? (Is it close?)**
      2. **How do you organize your stocks? Any methods to keep track of expiry dates, order of drugs?**
      3. **How often do you do inventory of your stores? Can you describe that process to me?**
      4. **How do you identify expired stock?**
      5. **Where do you put your expired drugs?**
      6. **How do you make sure your stock does not get damaged while in storage?**
   5. About how much time passes from receiving IFA until it is sent out to the Health Sub-Centers?
      1. Is distribution done at scheduled intervals? What are they?
      2. Connected with distribution of other goods/medications?
   6. **How is this process monitored? Are there any audits or evaluations done of this process?**
      1. **Who does them? How often?**
   7. **How were you trained logistics and supply managing?**
      1. **At beginning of this job? Were there additional trainings?**
      2. **Who gives the trainings? How often?**
      3. **Are they mandatory? Optional?**
      4. **Do you always attend these? If not, why?**
   8. **Is this you receive IFA in ALL blocks? Or do other blocks do it differently that you know of?**
3. From here, where is IFA sent?
   1. Who picks up the IFA?
   2. How often is IFA distributed from the primary health center?
      1. What frequency? (eg. every month?)
      2. When were they *last* distributed? How much was distributed? (# of pills / packs)
   3. How are IFA requests placed to the PHC?
      1. **Forms involved?**
      2. **Who submits these forms?**
      3. **Who do they submit the forms to?**
      4. How do ANMs request IFA if they run out before the next scheduled delivery? (*or do sub-centers make these requests?)*
   4. **How is this process monitored? Are there any audits or evaluations done of this process?**
      1. **Who does them? How often?**
   5. **How is the IFA distributed between ANMs, AWWs, and ASHAs? Can CDPOs or BHM/BCMs request IFA from you?**
      1. *Whose decision is this?*
      2. **Is there a policy that you follow when giving IFA out to these different officials or frontline workers?**
   6. When was the last time IFA supplements were not distributed on time?
      1. Can you describe to me what happened?
   7. **Do all PHCs distribute their IFA like this? Do they do it differently that you know of?**
   8. **Thinking of ALL the forms and procedures you just described which of these are on a computer registry? What computer systems are used? Which are done BY HAND?**
4. We’d also like to learn a little bit about how you decide how many iron and folic acid supplements are **needed** in your block.

*IFA Need*

- 1. What is the process that you go through to identify how many IFA supplements you need to request in your block?
     1. What data do you use to come to these estimates?
  2. Do you have or know where we could obtain these numbers **(for large (100mg) and small (20mg) pills)**??
     1. Number of IFA supplements needed in *your*  block
     2. Number of different pregnant women registered in ANC in block (last year, last month)
     3. Number of IFA supplements or packs administered in the last year (month) in the block.
     4. Number of IFA supplements or packs distributed from your block in the past year (month) from this primary health center.

1. **What POLICIES or GUIDELINES do you follow during receipt and distribution of drugs?**
   1. **What are they called?**
   2. **Could we SEE a copy?**
2. Would it be possible to see the iron and folic supplements that have not yet been distributed?
   1. [*Verify 100mg tabs and 20mg*] __________________
   2. [*Check expiration date*] __________________
   3. [*Note conditions of storage: climate controlled? Dry? How many are there?*] __________________
   4. **Could we see your stock inventory / registration documents? __________________**
3. **May we see the IFA receipt and distribution data from the last 2 years? Is this available?**
   1. ***______________________ IFA tablets requested: DATE ______________***
   2. ***_______________________ IFA tablets received: DATE ______________***
   3. ***_______________________ IFA tablets distributed to blocks: DATE ________***
   4. ***_______________________ IFA tablets to DH store: DATE________________***
4. Do you have any questions for us?
   1. Do you have any additional comments that you think we should know?
   2. **Is there anyone you would recommend us talking to in order to receive additional information on the IFA supply and distribution here?**

NAMES & CONTACT INFO: ____________________________________________________________________________________________________________________________________________________________________________________

Thank you so much for your time and participation today. It has helped is greatly in understanding the Iron and folic acid supplementation supply chain here in Bihar state. If we have further questions or inquiries about the IFA supply, would it be alright to contact you again?

**END TIME OF INTERVIEW _______:________ AM / PM**
