# Supplementary material for: Identifying bottlenecks in the iron and folic acid supply chain in Bihar, India: a mixed-methods study
Source: BMC Health Serv Res. 2018 Apr 12;18:281. doi: 10.1186/s12913-018-3017-x (PMC5898001; doi:10.1186/s12913-018-3017-x)
Supplement: Supplementary file 6 — IDI ANM: In-depth interview guide for Auxiliary Nurse Midwives. (DOCX 20 kb) [file 12913_2018_3017_MOESM6_ESM.docx]

*Due to the iterative and reflexive nature of qualitative research, this document served to guide the interviews with the participants and was not followed word for word. In some cases, questions may have been skipped, asked in a different order, or other questions added according to the participants’ responses and flow of the conversation.*

**IFA SUPPLY INTERVIEWS: ANM**

**START TIME OF INTERVIEW _______:________ AM / PM**

**INTRODUCTION**

We are interested in finding out what your experiences are receiving IFA from the PHCs, administrating it to pregnant women, and distributing it to other frontline workers. Our goal in this survey is not to evaluate your performance in any way but to understand the IFA supply situation and possible improvements that can be made.

1. Could you tell us a little about what your role is in giving IFA to pregnant women?
   1. When do you offer IFA to pregnant women?
2. We’re also interested in where the IFA that you receive comes from and how you distribute it to other frontline workers as well as to pregnant women.
   1. Could you walk is through the process of how you receive and distribute IFA?
   2. What changes happen if there is a delayed shipment or insufficient supply?
   3. *How is this process different for 20 mg IFA tablets?*
3. Now we’d like to understand the process of how you receive iron and folic acid tablets. [*May be covered in initial work flow diagram*]
   1. Who delivers the IFA to __________ the health sub-center? Or do YOU pick it up?
      1. Transport? (Delivery truck? Personal vehicle? Whose?)
   2. How often is the IFA delivered?
      1. What frequency?
      2. When were they *last* delivered?
      3. How much was received in the *last* delivery?
   3. How do you request deliveries? Please describe the process.
      1. **Forms involved**? **What does each form look like? (PHOTO OR COPY)**
      2. **Who is contacted**?
      3. What happens when you run out of IFA tablets before the next scheduled delivery?
         1. Same request process? Different? Is there one?
         2. Please describe this process if different.
            1. Different forms?
            2. Different people to contact?
   4. Where are shipments of IFA stored?
   5. Health Sub-Center? Other location? **Can we see this place? (Is it close?)**
   6. **How do you store your IFA? Why do you keep it there?**
   7. **Does anybody look over your records of pregnant women and IFA administration?**
      1. **Who does this? How often?**
      2. **What do they look for? How do they decide if you are doing well or not?**
      3. **Were you initially trained on keeping these records? When? By who?**
   8. **Were you trained on how to properly make an indent? Store your medicines?**
      1. **When was this training? Who offered it?**
      2. **Have there been any additional trainings about this?**
   9. About how much time passes from IFA receipt to distribution to the frontline workers?
      1. Is distribution done at scheduled intervals? What are they?
      2. Connected with distribution of other goods/medications? (eg. Medical kits?)
   10. **Is this how it works for all ANMs? Or do other ANMs do it differently that you know of? Do other districts or blocks distribute IFA to frontline workers differently that you know of?**
   11. About how much time passes from when you receive IFA from the PHC until you have administered all of it (to PW or FLWs)?
       1. Is there ever a time when you have no IFA?
   12. How do frontline workers request additional IFA?
       1. In what cases do you distribute IFA to ASHAs? Can you describe some specific times when you gave IFA to ASHAs? Why did they need it?
          1. For specific women?
          2. To replenish medical kit supply?
          3. Did they request it? How?
          4. Other?
       2. When do you distribute IFA to AWWs? Can you describe some specific times when you gave IFA to AWWs?
          1. For specific women?
          2. To replenish medical kit supply?
          3. Did they request it? How?
          4. Other?
   13. **Do other ICDS workers besides AWW have access to any 100mg IFA tablets?**
       1. **Can they request more for your village if they see a need?**
       2. **How much of the IFA supply (for pregnant women) comes through your supply for your area?**
       3. **Who decides how much IFA each frontline worker receives?**
   14. How is the receipt of IFA from the PHC documented? How do you document your distribution of IFA tablets to ASHAs? To AWWs?
       1. May we see this? (Take a photo – *outdoor register)*
   15. When was the last time you ran out of IFA and could not distribute it to FLWs?
       1. Can you describe to me what happened?
4. **What if three women came to you for IFA and at the time you only had enough IFA for one woman. How would you decide which women receive IFA and which ones don’t?**
5. I’d also like to learn more about how it’s decided how much IFA you need in order to have enough for the pregnant women in your area. How do you make sure the IFA requested is how much you need?
   1. How do you report pregnant women in your area? (How many in the last year / month)?
      1. **Can you SHOW us these registers?** *(Take pictures of the most recent pages mentioning IFA, if they don’t have IFA currently, then take pictures of the most recent record of when they had IFA)*
      2. **Are all of your records done by hand?**
   2. How do you report IFA administration to pregnant women? (How many IFA tablets administered in the last year / month?)
   3. Have you ever given IFA to a pregnant woman and not reported it? When did this happen?
   4. Which frontline workers in your area administer IFA to pregnant women?
      1. [*Ask about each profession mentioned*]:
      2. When do AWWs administer IFA to pregnant women?
         1. How do AWWs report IFA administration?
      3. When do ASHAs administer IFA to pregnant women?
         1. How do ASHAs report IFA administration?
   5. **Do you administer IFA to lactating women? Do you order enough IFA to administer to lactating women? Why (not)?**
      1. **Adolescents?**
      2. **Children?**
6. Would it be possible to see the iron and folic supplements that have not yet been distributed?
   1. [*Verify 100mg tabs, not 20mg*] __________________
   2. [*Check expiration date*] __________________
   3. [*Note conditions of storage: climate controlled? Dry? How many are there?*] __________________
7. Do you have any questions for us?
   1. Do you have any additional comments that you think we should know?
   2. **Is there anyone you would recommend us talking to in order to receive additional information on the IFA supply and distribution here?**

NAMES & CONTACT INFO: ____________________________________________________________________________________________________________________________________________________________________________________

Thank you so much for your time and participation today. It has helped is greatly in understanding the Iron and folic acid supplementation supply chain here in Bihar state. If we have further questions or inquiries about the IFA supply, would it be alright to contact you again?

**END TIME OF INTERVIEW _______:________ AM / PM**
