# Supplementary material for: Identifying bottlenecks in the iron and folic acid supply chain in Bihar, India: a mixed-methods study
Source: BMC Health Serv Res. 2018 Apr 12;18:281. doi: 10.1186/s12913-018-3017-x (PMC5898001; doi:10.1186/s12913-018-3017-x)
Supplement: Supplementary file 8 — IDI AWW: In-depth interview guide for Anganwadi workers. (DOCX 18 kb) [file 12913_2018_3017_MOESM8_ESM.docx]

*Due to the iterative and reflexive nature of qualitative research, this document served to guide the interviews with the participants and was not followed word for word. In some cases, questions may have been skipped, asked in a different order, or other questions added according to the participants’ responses and flow of the conversation.*

**IFA SUPPLY INTERVIEWS: AWW**

**START TIME OF INTERVIEW _______:________ AM / PM**

**INTRODUCTION**

1. We have heard that members of this community receive IFA. Could you tell me about who receives IFA? [*record all who receive IFA*]
   1. How do pregnant women receive IFA?
   2. What is your role in this?
   3. When do you offer IFA to pregnant women?
   4. **Do you offer IFA to lactating women? Why (not)?**
   5. **Is this how it works with all AWWs? Are some blocks different? Some districts?**
2. Could you tell me more about what you do when you give IFA to a pregnant woman?
   1. What do you say?
   2. What do you tell her about taking IFA?
   3. What do you tell her about nutrition during pregnancy?
      1. Iron consumption?
   4. Do you ask the woman if she is going to migrate? Is it required to ask this?
   5. If you are on a home visit, what else do you do on that visit besides IFA?
   6. **How many home visits do you do per month? ______ How many have you done in the past week? ________**
3. How do you receive IFA tablets that you give to pregnant women?
   1. Who do you receive them from?
   2. When do you usually get them?
      1. At regular intervals? (weekly, monthly?)
      2. Sporadically?
   3. How much do you usually receive?
      1. How long does this last you? (How long from receiving IFA do you run out of it?)
   4. Do you request IFA? If not, who decides how much you receive? Who decides when you receive IFA?
      1. If yes, how do you request them?
   5. Who decides where you offer them? Who do you offer them to?
4. **What if three women came to you for IFA and at the time you only had enough IFA for one woman. How would you decide which women receive IFA and which ones don’t?**
   1. What do you do if a woman asks for more IFA and you have none? What do you tell her?
5. Who else gives IFA to pregnant women?
   1. [*Ask about each profession mentioned*]:
   2. When do ANMs administer IFA to pregnant women?
   3. When do ASHAs administer IFA to pregnant women?
6. How do you track how many pregnant women are in your community? How many receive IFA?
   1. **Can you SHOW us these registers?** *(Take pictures of the most recent pages mentioning IFA, if they don’t have IFA currently, then take pictures of the most recent record when they had IFA)*
   2. ***Check for survey registry, pregnancy registry, due list registry***
   3. **How does this information get added to the MCH registry for the ANM?**
      1. **Who copies the information? (AWW or ANM?)**
   4. **Does anybody look over your registers? How often?**
   5. **Who trained you to do surveys? How to give out IFA?**
   6. **How often do you receive trainings?**
7. **Do you think there a demand in your community for IFA? Why (not)?**
8. Where do you store the IFA supplements you receive?
   1. Can we see the IFA pills that you have here?
      1. [*Verify 100mg tabs, not 20mg*] __________________
      2. [*Check expiration date*] __________________
      3. [*Note conditions of storage: climate controlled? Dry? How many are there?*] __________________
      4. [*Any visible forms / documentation for receipt or distribution? Describe.*]
9. Do you have any questions for us?
   1. Do you have any additional comments that you think we should know?
   2. **Is there anyone you would recommend us talking to in order to receive additional information on the IFA supply and distribution here?**

NAMES & CONTACT INFO: ____________________________________________________________________________________________________________________________________________________________________________________

Thank you so much for your time and participation today. It has helped is greatly in understanding the Iron and folic acid supplementation supply chain here in Bihar state. If we have further questions or inquiries about the IFA supply, would it be alright to contact you again?

**END TIME OF INTERVIEW _______:________ AM / PM**
