# Supplementary material for: Identifying bottlenecks in the iron and folic acid supply chain in Bihar, India: a mixed-methods study
Source: BMC Health Serv Res. 2018 Apr 12;18:281. doi: 10.1186/s12913-018-3017-x (PMC5898001; doi:10.1186/s12913-018-3017-x)
Supplement: Supplementary file 9 — Questionnaire ANM: Survey administered to Auxiliary Nurse Midwives. (DOCX 18 kb) [file 12913_2018_3017_MOESM9_ESM.docx]

**IFA SUPPLY QUESTIONNAIRE FOR ANM: TO BE GIVEN BY BLOCK COORDINATORS**

| IDENTIFICATION / LOCATION | |
| --- | --- |
| A. DATE OF INTERVIEW: _____________________________________  HEALTH SUB-CENTER ___________________________________________  BLOCK ______________________________________________________  DISTRICT _____________________________________________________  NAME OF THE INTERVIEWER ________________________________  DATE OF INTERVIEW _________________________________________ |  |

The purpose of this survey is to collect information on the status of IFA supply and protocols across the CARE focus districts. Information collected in this survey will not be used for evaluation of any person and will only be used to learn more about IFA supply status and protocols used at the ANM level. Participation in this survey is completely voluntary and you may refuse to answer any questions at any time. However, your answers and information in this will be invaluable and will enable us to successfully evaluate and understand the IFA supply chain as it exists in Bihar. Honest responses especially concerning the reality in the field will be sincerely appreciated and most helpful in determining bottlenecks of the system and possibilities for improvement.

1. How many people does your health sub-center cover?
   1. ___________________ (number of people)
2. What is your stock of large IFA tablets (100mg) right now?
   1. Number of Tablets: _________________________
3. How often do you receive large (100mg) IFA tablets?
   1. Approximately every ___________ months
   2. Not fixed
4. When did you last request large (100mg) IFA tablets?
   1. Date: ___________________
5. When did you receive your last delivery of large (100mg) IFA tablets?
   1. Date: ___________________
6. How many large (100mg) IFA tablets did you last receive?
   1. Number of tablets: ________________________
7. Do you receive IFA on a regular basis or do you have to request IFA?
   1. I receive IFA through fixed delivery times and can request if needed.
   2. I receive IFA through fixed deliveries and cannot request IFA
   3. I can only receive IFA through request
8. Who do you approach to ask for more IFA?
   1. ________________________ (title of person).
9. How do you request more IFA tablets?
   1. Verbally.
   2. Written.
   3. I don’t request more IFA tablets.
10. Who approves and verifies your request?
11. No one
12. ……………………….approves
13. ………………………..verifies
14. When do you request additional IFA tablets?
    1. I request additional IFA when stocks reach ___________ (# of pills).
    2. I request additional IFA only when I am completely out of IFA.
    3. I cannot request IFA.
15. If you request IFA tablets, how much do you usually request?
    1. ____________ IFA tablets to last _______________ months
    2. The number varies greatly, last time I ordered: ___________ IFA tablets to last ___________ months
16. If you request IFA tablets, how much do you receive from your request?
    1. The full amount requested
    2. Partial amount
    3. I rarely / never receive any amount of IFA that I request
17. If you request IFA tablets, how long does it take for your requested IFA to arrive?
    1. ………………….. weeks
    2. …………………... months
18. How do you decide how much IFA you need?
    1. Based on population and pregnancy rates
    2. Based on how much IFA is used
    3. Based on number of pregnant women registered for ANC
    4. Other rationale: _____________________
19. Besides the PHC store, where else do you get IFA if you need it?
    1. I can take from ASHA kits in my area
    2. I can go to other sub-centers and borrow their IFA
    3. I can purchase IFA using sub-center funds if there is a need
    4. If there is no IFA at the PHC, I cannot get it anywhere and cannot distribute it
    5. Other way: _______________________________________
20. How many large (100mg) IFA tablets do you administer to the following in an average month:
    1. AWW: ____________ (# of pills)
    2. ASHA: ____________ (# of pills)
    3. Pregnant Women: _______________ (# of pills)
    4. Lactating Women: _______________ (# of pills)
    5. Other ______________: _____________ (# of pills)
21. How many pregnant women have you registered in the past 3 months?
    1. ________________
22. How pregnant women have you administered IFA to in the past 3 months?
    1. ________________
23. How many IFA tablets have you administered to pregnant women in the past 3 months?
    1. ________________
24. Do you ask women who you register for ANC if they are planning to migrate to another place during pregnancy?
    1. Yes – **If yes, go to questions #22 and #23**
    2. No – **If no, skip to question #24**
25. If she is migrating, do you change how you give the woman IFA?
    1. No.
    2. Yes, I give her more IFA. - **Skip to question #23**
    3. Yes, I give her all 100 IFA at her first ANC visit. – **Skip to question #23**
    4. Other action: _____________________________________________
26. If no, how do you make sure the woman receives all of her IFA?
    1. I tell her to go to the PHC or AWC for IFA when she arrives to her destination.
    2. The AWW or ASHA will find her doing home visits and give her IFA, I do not have to do anything.
    3. She is not in my population anymore so she is not my responsibility.
    4. Other action: _______________________________________________
27. Do you have any small (20mg) IFA tablets right now?
    1. Yes
    2. No – **Skip questions #23-28, Go to #29**
28. How many small (20mg) IFA tablets do you have right now?
    1. Number of tablets: _________________
29. How often do you receive small IFA tablets?
    1. Every __________ months
    2. Not fixed
30. When did you last request small IFA tablets?
    1. Date: ______________________
31. When did you last receive small IFA tablets?
    1. Date: ______________________
32. How many small IFA tablets did you last receive?
    1. Number of tablets: ______________________
33. How many small IFA tablets do you usually administer in one month?
    1. AWW: ____________ (# of pills)
    2. ASHA: ____________ (# of pills)
    3. Children ____________ (age group) : _______________ (# of pills)
    4. Other ______________: _____________ (# of pills)
34. Do you have any IFA syrup right now?
    1. Yes
    2. No – **End interview**
35. How many bottles of IFA syrup do you have right now?
    1. Number of bottles: _________________
36. How often do you receive IFA syrup?
    1. Every __________ months
    2. Not fixed
37. When did you last request IFA syrup?
    1. Date: ______________________
38. When did you last receive IFA syrup?
    1. Date: ______________________
39. How many IFA syrup bottles did you last receive?
    1. Number of bottles: ______________________
40. How many IFA syrup bottles do you usually administer in one month?
    1. AWW: ____________ (# of bottles)
    2. ASHA: ____________ (# of bottles)
    3. Children ____________ (age group) : _______________ (# of bottles)
    4. Lactating women: ____________________ (# of bottles)
    5. Other ______________: _____________ (# of bottles)
